# Miscellaneous

**Published:** 1891-01

**Authors:** 


### MISCELLANEOUS.

### CAST IRON BRICKS.

What are termed hollow cast iron bricks form the subject of a recent German patent described in the technical journals, the article being the invention of an Erfurt mechanic. As the name indicates, they are made of regular brick form and size, the walls being 0.12 inches thick, but no mortar or other binding material is intended to enter into their use, the method of fastening adopted being as follows : The upper and lower sides of the bricks are provided with grooves and protecting ribs, which fit into one another easily and perfectly, so as to make a uniform and complete union or combination.

There are in addition two large circular openings in the upper side of each brick, arranged to receive suitably formed projections on the lower side of the brick above, one of these projections being also hooked shape, thus securing a more secure hold ; and in order that the joints be made and remain air and water tight, a fluid is applied to the surface of the bricks with a brush. The non-conducting air spaces in the bricks, and the ease with which they may be put together and taken apart without injuring them, are cited as special advantages in their favor as a substitute for ordinary bricks and brick construction.

---

A Living Emetic.—A servant who did not find her way very promptly to the kitchen one morning was visited by her mistress, who found her in bed, suffering from pain and violent sickness. She explained that she had a cold and had taken some medicine which had been recommended for the children. "How much did you take ?" asked her mistress. "Well, mum,I went by the directions on the bottle. It said : 'Ten drops for an infant, thirty drops for an adult and a tablespoonful for an emetic.' I knew I wasn't an infant or adult, so I thought I must be an emetic, and the pesky stuff has pretty nigh turned me insides out."—*Medical Brief.*

Statistics show that ninety-five out of a hundred men fail in business sooner or later, and the cases in which a firm sees fifty years of business life are extremely rare. It is certainly then a noteworthy case when a house dates its existence back to the close of the Revolution, as do Walter Baker & Co., the famous chocolate and cocoa manufacturers of Dorchester, Mass., who began business there in 1780, and for a hundred and ten years have made their productions the standard of purity and excellence all over the world. The immense increase in the consumption of their Breakfast Cocoa is largely due to their sagacity in setting and maintaining the standard of absolute purity in its production, thereby insuring its perfect healthfulness and the highest degree of nutrition. No chemicals are ever used in its preparation, but only the action of the cleanest and most exact mechanical processes upon the best materials ; and at the Paris Exposition the gold medal for absolute purity and excellence was awarded to W. Baker & Co.'s preparations by the most eminent scientific authorities of Europe.

### HARVEY'S WONDERFUL POWER.

A young printer employed by the Blakely Printing Company is astonishing his associates with some marvelous feats, says the *Farmer and Exchange*. The accuracy with which he takes measurements with his eyes and mind seems really supernatural. In using pica measure—a pica is one-sixth of an inch—he employs no guide to aid him, but adjusts his stick, cuts rules and leads the desired length by measuring the space in his mind. In giving an exhibition of his powers lately, he took nine pieces of brass rule and cut them to 6, 7, 8, 9, 10, 11, 12, 13, 14 and 15 pica ems respectively, and on measuring the cut rules they were found to be the exact length. He then cut twelve pieces to one length without comparing them. The pieces were gathered and compared and could not have been shaved nearer one length. He tells longer measures with equal accuracy. A piece of wood was handed him and he was asked the length of it. "Two feet five and three-eighth inches," was the quick and absolutely accurate reply. He was also asked if a certain block was square. "One of its angles lacks a little of it," he answered. The angle referred to was found to be slightly acute. Another curious freak of this printer is that he never carries a watch, but is a positive regulator at telling the hour. One dark afternoon, after having not seen a clock all day, one of the men jestingly asked him the time of day. "Seven minutes after 3," was the prompt reply, and all the correct watches in the house testified to his accuracy. To more fully test his ability in this direction, his room-mate awoke him a few nights ago and put to him the query as to the hour. The reply this time was "twenty minutes after 2," which was correct to a minute. The young printer dislikes notoriety and refuses to use his peculiar gift publicly for gain. His name is Joe Harvey. Since a boy he has been able to accomplish remarkable feats of this kind.

---

Hon. George Bancroft, the historian, spent his ninetieth birthday, October 3d, in his Newport cottage on the "Cliff," which was thronged with callers. He is still an untiring reader. In 1882, Mr. Bancroft wrote thus to S. Austin Allibone: "I was trained to look upon life here as a season of labor. Being more than four score years old I know the time for my release will soon come. Conscious of being near the shore of eternity, I await without impatience and without dread the beckoning of the hand which will summon me to rest." In his long and active life Mr. Bancroft has been Collector of the Port of Boston under President Van Buren, Secretary of the Navy under Polk, and acted as Secretary of War for a month at the same time, Minister to Great Britain, Prussia, the North German Confederation and the German Empire. He published the first volume of his history of the United States in 1834 and the last in 1874. Some revisions have been published, the last in 1885.

It has been found that, by the addition of chloride of zinc to the pulp, in course of manufacture, paper may be made as tough as wood or leather; the degree of concentration in the zinc solution determining its toughness. This substance can be utilized in a variety of ways, as it is thoroughly waterproof.

### DINING IN LAPLAND.

I was taken into one of the Lapp's huts, says a writer in an English journal. In the centre a wood fire was burning brightly on some stones, and at first the smoke was very unpleasant, but soon one became accustomed to it, and it served the useful purpose of driving away the winged plague, which had followed us all day.

The man proceeded to boil some coffee, which in a few minutes was set before me, together with a wooden bowlful of reindeer's milk. The coffee was not very palatable, but under the circumstances worse fare would have proved acceptable. The milk I found to be too thick and rich to drink much of.

A sugar loaf was produced from beneath some cloths in a corner, and a few pieces were chipped off and handed to me. I accepted them with my politest smile, accompanied by a bow, but when I proceeded to sugar my coffee in the orthodox style the action caused much amusement to the juvenile Lapps, who roared with laughter, and appeared to enjoy the fun immensely.

I found that I ought to have eaten the sugar separately, as they did, and they evidently considered my way of sweetening coffee inexpressibly funny.

Cakes were then served to each one. These were about the size of a penny bun, but of the consistency of putty or dough, which they somewhat resemble in appearance. Sour cream was eaten with them. So nasty were they that a mouthful gave me "quite a turn," and I was glad to smuggle the remainder underneath the rug on which I was sitting. I did not like to throw it away, for fear of offending my hosts, but trusted to the sharp noses of the dogs to get me out of the difficulty.

---

Characteristics of Handwriting.—Handwriting has its characteristics, and is a study in itself to those who want to become familiar with its peculiarities, says the St. Louis *Globe-Democrat*. It can very easily be told whether a person whose writing you want to identify is a man or a woman, a minor or adult. It is very seldom a handwriting assumes its permanency before the writer is twenty-five years old. The age of the writing can approximately be determined by various methods. If it has a Spencerian appearance you may know it was written after 1882, as at that date the Spencerian system was introduced. If it is the black aniline ink that is generally used everywhere now, you may know it was written after 1873. The older inks had iron or some diluted dyestuff for a basis, and preceded the aniline. An analysis of the writing will most generally determine the date of the writing.

For Croup use kerosene oil. Wet a piece of flannel and apply. It gives almost instant relief. Remove when the skin becomes very red, or it will blister.

Care of Mad Dog Bites.—A German forest keeper recommends a cure for the bite of a rabid animal, which is being widely circulated in the newspapers. It consists of bathing the wound with vinegar and pouring upon it a few drops of muriatic acid. We are sure that any one who tries this remedy will find it nearly as bad as the bite. Muriatic acid is a powerful corrosive.

### THE MURDEROUS CIGARETTES.

The *Chicago Herald* gives an account of a young man, residing in that city who recently became insane through cigarette smoking. The young man began the use of cigarettes about a year ago. Cases of insanity from the use of cigarettes are becoming more and more frequent.

At Lockport, N. Y., a boy named Fred. Long, aged fourteen years, recently died from heart disease, superinduced by cigarette smoking, and this is only one in thousands.

